# Schizophrenia and Hospital Admissions for Cardiovascular Events in a Large Population: The APNA Study

**DOI:** 10.3390/jcdd9010025

**Published:** 2022-01-13

**Authors:** Sara Guillen-Aguinaga, Antonio Brugos-Larumbe, Laura Guillen-Aguinaga, Felipe Ortuño, Francisco Guillen-Grima, Luis Forga, Ines Aguinaga-Ontoso

**Affiliations:** 1 Azpilagaña Health Center, Navarra Health Service, 31006 Pamplona, Navarra, Spain; sguillen.4@alumni.unav.es; 2 Department of Health Sciences, Public University of Navarra (UPNA), 31008 Pamplona, Navarra, Spain; ablm649@gmail.com (A.B.-L.); ines.aguinaga@unavarra.es (I.A.-O.); 3 Department of Nursing, Clinica Universidad de Navarra, 31008 Pamplona, Navarra, Spain; lguillen@unav.es; 4 Department of Psychiatry, Clinica Universidad de Navarra, 31008 Pamplona, Navarra, Spain; fortunos@unav.es; 5 Navarra Institute of Health Research (IdiSNA), 31008 Pamplona, Navarra, Spain; lluis.forga.llenas@navarra.es; 6 Department of Preventive Medicine, Clinica Universidad de Navarra, 31008 Pamplona, Navarra, Spain; 7 CIBER-OBN, Instituto de Salud Carlos III, 28029 Madrid, Comunidad de Madrid, Spain; 8 Department of Endocrinology, University Hospital of Navarra, C/Irunlarrea s/n, 31008 Pamplona, Navarra, Spain

**Keywords:** cardiovascular, schizophrenia, prospective cohort, hospital admissions

## Abstract

(1) Background: Patients with schizophrenia have higher mortality, with cardiovascular diseases being the first cause of mortality. This study aims to estimate the excess risk of hospital admission for cardiovascular events in schizophrenic patients, adjusting for comorbidity and risk factors. (2) Methods: The APNA study is a dynamic prospective cohort of all residents in Navarra, Spain. A total of 505,889 people over 18 years old were followed for five years. The endpoint was hospital admissions for a cardiovascular event. Direct Acyclic Graphs (DAG) and Cox regression were used. (3) Results: Schizophrenic patients had a Hazard Ratio (HR) of 1.414 (95% CI 1.031–1.938) of hospital admission for a cardiovascular event after adjusting for age, sex, hypertension, type 2 diabetes, dyslipidemia, smoking, low income, obesity, antecedents of cardiovascular disease, and smoking. In non-adherent to antipsychotic treatment schizophrenia patients, the HR was 2.232 (95% CI 1.267–3.933). (4) Conclusions: Patients with schizophrenia have a higher risk of hospital admission for cardiovascular events than persons with the same risk factors without schizophrenia. Primary care nursing interventions should monitor these patients and reduce cardiovascular risk factors.

## 1. Introduction

Schizophrenia is a severe mental illness associated with elevated cardiovascular disease risk. Patients with schizophrenia have higher comorbidity (notably higher risk of cardiovascular diseases) and higher mortality [1]. A meta-analysis showed that mortality rates were 2.5 times higher in patients with schizophrenia. Suicides were higher in patients with schizophrenia. In addition, mortality rates from the major causes of death were higher in patients with schizophrenia than in the general population [2]. Cardiovascular disease is the first leading cause of mortality in patients with schizophrenia, followed by cancer and suicide [3,4].

Likewise, the onset of the first episode of vascular diseases is lower [5]. Moreover, revascularization rates are lower after an infarction in patients with schizophrenia [6]. A cohort study conducted in Singapore showed that patients with schizophrenia with physical comorbidity (diabetes, hypertension, coronary heart disease, and cancer) had an increased mortality risk [7]. A problem in patients with schizophrenia is non-adherence to treatment with antipsychotic drugs, which can reach 42% [8]. However, this figure could be even higher because a clinical trial found 74% of patients discontinued antipsychotic treatment after 1.5 years [9]. This non-adherence could affect lifestyle and affect the appearance of cardiovascular diseases [10,11,12,13].

This research aims to study whether patients with schizophrenia have an increased risk of hospital admissions for cardiovascular diseases independently of their comorbidity.

## 2. Materials and Methods

The “Navarre primary health care system cohort” (APNA study) is a dynamical multipurpose prospective cohort. It started in 2004 in the autonomous community of Navarre (northern Spain). Details on the cohort design are available elsewhere [14,15,16,17,18,19].

Briefly, the population of Navarra primary health care data has been recorded since 2004 [17]. In this study, we selected people over 18 years old. The follow-up in this study was from 2012 to 2016. The average population in the period was 505,889 people.

### 2.1. Study Variables

We collected age, sex, date of hospital admissions for cardiovascular events, and income status. Income levels were obtained from the TSI used for the pharmaceutical co-payment. TSI 001 code which includes people with non-contributory pensions (5150 EUR/year), Social Integration Income (7640 EUR/year), or unemployed people without unemployment benefits, was considered low income [20]. We obtained from the Navarra hospital discharge registration system the date of the first hospital admission with a cardiovascular event (CVE) as the main reason for admission during the follow-up period. We considered CVE those hospital admissions with a diagnosis codified as a cerebrovascular disease with the International Classification of Diseases 10th version (ICD10) codes 430–438; ICD10: I60–I69) or ischemic heart (ICD10: 410–414; ICD10: I20–I25). We consider as an antecedent of CVE a hospital admission with a diagnosis codified as cerebrovascular disease or ischemic heart disease before 2012. We followed patients until an event occurred or until 31 December 2016.

Clinical variables: the following variables were used: weight (kilograms), height (meters), Body Mass Index (BMI) (weight/height^2^), systolic and diastolic BP (mmHg), and smoking during the follow-up period. Patients with BMI ≥ 30 were classified as obese. We recorded diagnoses of type 2 diabetes, hypertension, and dyslipidemia. We extracted the antecedents of previous hospital admission for cardiovascular events from hospital records. We used the same codes mentioned above. Comorbidity was computed with the Charlson Index [21] and the Comorbidity Index [22].

Patients with schizophrenia diagnosis using ICD10 by a psychiatrist from Navarre Health Service were identified in the database. A person who had not been diagnosed with schizophrenia was considered free from the illness.

The number of bottles of antipsychotic medication purchased at each pharmacy by each patient was extracted from the drug prescription database. A patient was considered non-adherent to antipsychotic treatment when they had not purchased antipsychotic medication from the pharmacy for one year. The definition of non-adherence to antipsychotic treatment is much debated [23,24]. In general, non-adherence to antipsychotic treatment exists when patients stop taking between 20% and 30% of the medication [23,24]. In our case, we did not have monthly figures and only annual figures, so it was difficult to know if a patient had stopped taking the medication since there could be two scenarios: a patient could change medication or take several medications simultaneously. For this reason, we chose one year without any medication as the criterion for non-adherence.

### 2.2. Statistical Analysis

Although it is helpful for simple causal structures, the traditional approach to confounding is inadequate for more complex causal networks. Adjustments for many variables can open back paths and introduce bias [25]. For these reasons, many editors agreed in a document entitled “Guidelines for the Control of Confounding and Reporting of Results in Causal Inference Studies” [25].

A valuable tool for this purpose is DAGs (Directed Acyclic Graph). DAGs allow drawing a theoretical map of the relationships among variables determining what variables should be controlled in a multivariate model, avoiding bias [26]. In a DAG, unidirectional arrows represent known causal effects (based on the existing knowledge). DAG allows determining what variables should be controlled as cofounders. The determination of the variables to be controlled can be done manually or using software such as Daggity [27].

**Figure 1:**
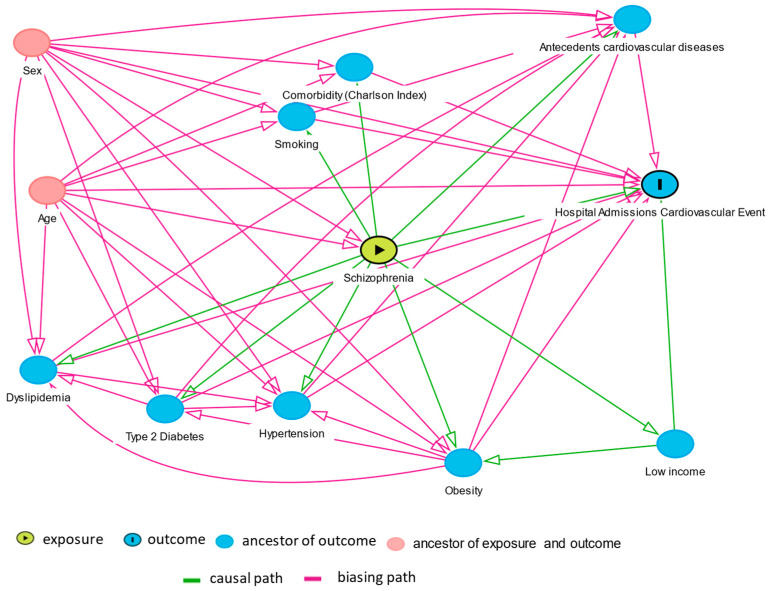
Directed Acyclic Graph (DAG) of schizophrenia and hospital admissions for cardiovascular events.

We used DAG (Figure 1) to study the relationship of schizophrenia with hospital admissions for cardiovascular events and potential confounding factors. The DAG graphics indicates that sex influences dyslipidemia, type 2 diabetes, smoking, Charlson Index, hypertension obesity, antecedents of cardiovascular events, schizophrenia, and hospital admissions for cardiovascular events. Using this graph, the software Dagitty indicated that the possible cofounders that should be adjusted were age, antecedents cardiovascular diseases, comorbidity (Charlson Index), dyslipidemia, hypertension, income, obesity, sex, smoking, and type 2 diabetes. Supplementary Figure S1 shows the causal path between schizophrenia and hospital admissions for cardiovascular events.

In the descriptive analysis, we present the data of categorical variables as proportion with confidence intervals. When adequate, we give mean and standard deviation or median and interquartile deviation in the quantitative variables. Comparisons among categorical variables were calculated with the Chi-square test. Comparisons between groups in quantitative variables were performed with Student’s *t*-test. When quantitative variables were scales or had no normal distribution, the Kruskal–Wallis test was performed.

Multivariate analysis was performed in two steps. First, a DAG was drawn. Daggity software identified the variables to be adjusted. In the second step, the multivariate models were made adjusting for those variables identified as cofounders in the DAG. Cox regression was used to estimate the excess risk of hospital admissions for cardiovascular events associated with schizophrenia. In the Cox models, adjustment was made for all variables identified by as confounding factors in the DAG. There was a high correlation between Charlson and the Comorbidity Index. As a result, we used the Charlson Index in the analysis. Data were analyzed with IBM SPSS and Openepi [28].

## 3. Results

The analysis included 505,889 people with 2,700,505 follow-up years. During the following period, there were 8639 vascular events. The incidence of cardiovascular events was 355.5 per 100,000 person-years in patients with schizophrenia versus 91.1 per 100,000 person-years in persons without schizophrenia with a Rate Ratio of 3.903 (95% CI 2.924–5.208). The proportion of males was higher in persons with schizophrenia, 59.8%, than in persons without schizophrenia, 49.2% (Table 1). The proportion of persons with low income was four times higher in patients with schizophrenia, 12.9% vs. 3.8%. Obesity, diabetes, and smoking were higher in patients with schizophrenia, while the prevalence of hypertension was lower than in persons without the disease. Dyslipidemia, comorbidity, and Charlson Index were similar in both groups.

There were 2495 patients with schizophrenia. Patients with schizophrenia had been diagnosed for an average of 15.82 years. (95% CI 14.19–17.45). Of these patients, 9.5% had antecedents of cardiovascular events.

The more common treatment was Oxazepine and Thiazepine, with 39.5% of the patients taking the medication, followed by Risperidone 19.3% and Olanzepine with 18.1% (Table 2).

A moderate percentage of patients with schizophrenia, 27.77% (95% CI 26.04–29.56), were non-adherent to antipsychotic treatment, i.e., they had not withdrawn their prescription medication from the pharmacy for at least one year.

The Charlson Index was not included in the models because of collinearity with age. The models with the Charlson Index are presented in the supplementary material (Tables S1 and S2). The results are very similar to those without the Charlson Index presented in the article.

On the Cox model adjusted for age and sex, patients with schizophrenia compared with people without schizophrenia had a Hazard Ratio (HR) of 1.348 (1.009–1.801) for a cardiovascular event hospital admission, *p* = 0.044 (Table 3). In the final multivariate model adjusted for age, sex, hypertension, type 2 diabetes, dyslipidemia, income, obesity, antecedents of cardiovascular disease, and smoking, patients with schizophrenia had an HR of 1.421 (95% CI 1.037–1.948).

Patients with schizophrenia who have stopped treatment had an HR adjusted by age and sex of 1.778 (95% CI 1.071–2.951). There were no differences in the risk between patients with schizophrenia following antipsychotic treatment and those without schizophrenia (Table 4).

The final multivariate model was adjusted for age, sex, hypertension, type 2 diabetes, dyslipidemia, smoking, low income, obesity, antecedents of cardiovascular events, and smoking. Patients with schizophrenia without treatment had an HR of 2.232 (95% CI 1.267–3.933). The most significant risk factor for developing a cardiovascular event was having had another cardiovascular event that required hospitalization prior to the follow-up period, with an HR of 6.559, followed by non-adherence to antipsychotic treatment with an HR of 2.232, followed by male sex with an HR of 1.953, type 2 diabetes with an HR of 1.531, and low income with 1.494. In conclusion, we can state that non-adherence to antipsychotic treatment increases the risk of having a cardiovascular event that requires hospitalization by 123%.

## 4. Discussion

This study shows that people with schizophrenia have a higher probability of cardiovascular events. This association remains after adjusting for sociodemographic factors and comorbidity. Individuals with severe mental illness experience increased morbidity and mortality compared to the general population. Among the explanations could be a higher prevalence of hazardous health behaviors that are cardiovascular risk factors (smoking, addiction, poor diet, lack of exercise, obesity) [29]. Molecular and genetic mechanisms [30], such as an accelerated shortening of the telomeres, may also play a role [31].

It is also possible that there may be differences in the accessibility of health care, the detection of somatic diseases, their treatment, and the attitudes of health care professionals [32]. A metanalysis found that patients with schizophrenia have less screening and lower-quality treatment [33]. Thus, a retrospective cohort study conducted in Ontario showed that patients with schizophrenia were less frequently treated with thrombolysis. These patients had fewer diagnostic tests, such as carotid imaging. They were less likely to be in rehabilitation or treated with anticoagulants, antihypertensives, or lipid-lowering drugs at discharge [34].

The cardio-metabolic adverse effects of antipsychotics, including weight gain, may contribute to the development of metabolic syndrome [3,35,36], which is associated with an increased risk of all-cause and cardiovascular disease mortality. Antipsychotic drugs may exacerbate atherosclerosis [37]. Dysregulation of coagulation and complement may occur in patients with schizophrenia under treatment [38]. However, we found that patients who had discontinued antipsychotic treatment had a higher risk of cardiovascular events.

Patients with schizophrenia with diabetes have a higher risk of mortality and cardiovascular disease than persons without schizophrenia with diabetes [39]. Although the mechanisms leading to increased morbidity from cardiovascular disease are not yet precise, clinicians, especially general practitioners (GPs) dealing with patients with schizophrenia, should be aware that they have an increased likelihood of cardiovascular disease. In this research, we adjusted for the factors influencing CVE. The increased CV risk may be due to factors that have not been assessed for the degree of control of these risk factors, such as diabetes, hypertension, and dyslipidemia. Our investigation has adjusted for being diabetic or not, but not for being well or poorly controlled for diabetes. Future research should study differences in the control of cardiovascular risk factors.

Nurses’ intervention programs have been shown to reduce cardiovascular risk factors in women with schizophrenia [40]. Clinical trials are currently underway to reduce cardiovascular risk in young patients with schizophrenia [41]. Nurses’ health promotion programs should be carried out in patients with schizophrenia to reduce cardiovascular risk factors [42]. Another issue we detected is that patients who do not follow the treatment have a higher risk of developing a cardiovascular event. The explanation could be because they changed their lifestyle to a higher-risk lifestyle. In any case, another intervention of the community nurse would be to follow up with these patients to ensure that they take their medication.

In this study, the main limitation is that we only had access to hospital admissions. We did not have access to death certificates, so the incidence rate is probably underestimated and higher than the estimates presented. Another limitation is that we did not analyze the physical exercise of the patients since this is an item that is usually poorly recorded in the computerized medical records. One limitation is that the medication information we handle only tells us that the antipsychotic medication was purchased by the patient or the patient’s family, but we do not know whether the patient was taking the medication. Our study provides lower figures for non-adherence to antipsychotic treatment than those of the literature because our criterion was very stringent: one year without picking up the medication from the pharmacy, which could lead to underestimating non-adherence to antipsychotic treatment. The figures in the literature are slightly higher than ours. A meta-analysis found around 42% non-adherence to antipsychotic treatment compared to the figure we found of 27.77% [8]. However, it is more likely that lifestyle changes affect CVE when the period without medication is more than one year versus three months in the most common definitions.

## 5. Conclusions

Schizophrenia patients have a higher risk of hospital admission for cardiovascular events than persons with the same risk factors without schizophrenia. Specialist physicians and general practitioners need to be aware of this increased cardiovascular risk in patients with schizophrenia and instruct nurses to monitor them and include them in their follow-up programs. Primary care nursing interventions should monitor these patients and reduce cardiovascular risk factors.

## Figures and Tables

**Table 1:** Patients’ characteristics.

	Schizophrenia N = 2495	No Schizophrenia N = 503,394	
Variables	% Mean (SD)	% Mean (SD)	*p*
Age (years)	49.7 (15.7)	50.0 (18.0)	0.342 ^†^
Male	59.8%	49.2%	<0.001 ^‡^
Low income	12.9%	3.8%	<0.001 ^‡^
Charlson Index	1.3 (1.8)	1.3 (1.7)	0.677 *
Comorbidity Index	1.2 (1.5)	1.2 (1.7)	0.512 *
(Obesity) BMI ≥ 30	41.5%	25.9%	<0.001 ^‡^
BMI	29.4 (6.3)	27.2 (5.2)	<0.001 ^†^
CV antecedents	8.9%	9.5%	0.243 ^‡^
Diabetes	7.8%	5.4%	<0.001 ^‡^
Hypertension	12.5%	15.1%	0.001 ^‡^
Dyslipidemia	27.1%	25.9%	0.221 ^‡^
Smoking	19.8%	9.1%	<0.001 ^‡^

^†^ Student’s T; ^‡^ Chi-square; * Kruskal–Wallis; SD = standard deviation; BMI = Body Mass Index; CV = cardiovascular.

**Table 2:** Distribution of patient antipsychotic medication.

Medications	N	% *
Oxazepine and thiazepine	984	39.5
Risperidone	482	19.3
Olanzepine	451	18.1
Clozapine	264	10.6
Quetiapine	238	9.5
Clotiapine	128	5.1
Asenapine	8	0.3

* The percentages add up to more than 100% because patients may take two drugs or change medications.

**Table 3:** Cox regression for hospital admission for cardiovascular events in patients with schizophrenia.

	Model Adjusted by Age and Sex		Model Adjusted *	
Variables	HR (95% CI)	*p*	HR (95% CI)	*p*
Schizophrenia	1.348 (1.009–1.801)	0.044	1.421 (1.037–1.948)	0.029

HR = Hazard Ratio; * adjusted by hypertension, age, sex, type 2 diabetes, dyslipidemia, cardiovascular antecedents, smoking, income, and obesity (BMI ≥ 30).

**Table 4:** Cox regression for hospital admission for cardiovascular events according to schizophrenia treatment.

	Model Adjusted by Age and Sex		Full Model Adjusted *	
Variables	HR (95% CI)	*p*	HR (95% CI)	*p*
No schizophrenia	1	-	1	-
Treated schizophrenia	1.206 (0.848–1.717)	0.297	1.224 (0.838–1.267)	0.296
Non-adherence to antipsychotic treatment	1.778 (1.071–2.951)	0.026	2.232 (1.267–3.933)	0.005
Age	1.077 (1.076–1.079)	<0.001	1.044 (1.042–1.046))	<0.001
Sex (male)	2.465 (2.355–2.580)	<0.001	1.953 (1.857–2.054)	<0.001
High blood pressure	-	-	1.143 (1.076–1.189)	<0.001
Type 2 diabetes	-	-	1.531 (1.451–1.615)	<0.001
Antecedents CVE	-	-	6.559 (6.218–6.919)	<0.001
Smoking	-	-	1.134 (1.046–1.230)	0.002
Low income	-	-	1.494 (1.307–1.709)	<0.001
Dyslipidemia diagnosis or total cholesterol > 250 mg/dL	-	-	1.080 (1.030–1.132)	0.001
Obesity BMI ≥ 30	-	-	1.145 (1.090–1.203)	<0.001

HR = Hazard Ratio; CVE = cardiovascular event; BMI = Body Mass Index; * adjusted by all the variables in the model.

## Data Availability

The datasets generated for this study are unavailable due to the data protection law.

## Supplementary Materials

The following supporting information can be downloaded at: https://www.mdpi.com/article/10.3390/jcdd9010025/s1. Table S1: Univariate and multivariate cox regression for hospital admission for cardiovascular events adjusted by Charlson Index; Table S2: Univariate and multivariate cox regression for hospital admission for cardiovascular events according to adherence to schizophrenia treatment adjusted by Charlson Index; Figure S1: DAG showing the causal paths from schizophrenia to hospital admission for cardiovascular events.

## References

[1] Lavagnino L., Gurguis C., Lane S. (2021). Risk Factors for Metabolic and Cardiovascular Disease in Inpatients with Severe Mental Illness. Psychiatry Res..

[2] Saha S., Chant D., McGrath J. (2007). A Systematic Review of Mortality in Schizophrenia: Is the Differential Mortality Gap Worsening over Time?. Arch. Gen. Psychiatry.

[3] Azad M.C., Shoesmith W.D., Al Mamun M., Abdullah A.F., Naing D.K.S., Phanindranath M., Turin T.C. (2016). Cardiovascular Diseases among Patients with Schizophrenia. Asian J. Psychiatr..

[4] Moreno-Küstner B., Guzman-Parra J., Pardo Y., Sanchidrián Y., Díaz-Ruiz S., Mayoral-Cleries F. (2021). Excess Mortality in Patients with Schizophrenia Spectrum Disorders in Malaga (Spain): A Cohort Study. Epidemiol. Psychiatr. Sci..

[5] Marche J.-C., Bannay A., Baillot S., Dauriac-Le Masson V., Leveque P., Schmitt C., Laprévote V., Schwan R., Dobre D. (2021). Prevalence of Severe Cardiovascular Disease in Patients with Schizophrenia. Encephale..

[6] Fleetwood K., Wild S.H., Smith D.J., Mercer S.W., Licence K., Sudlow C.L.M., Jackson C.A. (2021). Severe Mental Illness and Mortality and Coronary Revascularisation Following a Myocardial Infarction: A Retrospective Cohort Study. BMC Med..

[7] Tan X.W., Lee E.S., Toh M.P.H.S., Lum A.W.M., Seah D.E.J., Leong K.P., Chan C.Y.W., Fung D.S.S., Tor P.C. (2021). Comparison of Mental-Physical Comorbidity, Risk of Death and Mortality among Patients with Mental Disorders—A Retrospective Cohort Study. J. Psychiatr. Res..

[8] Cramer J.A., Rosenheck R. (1998). Compliance With Medication Regimens for Mental and Physical Disorders. Psychiatr. Serv..

[9] Xiao J., Huang J., Long Y., Wang X., Wang Y., Yang Y., Hei G., Sun M., Zhao J., Li L. (2021). Optimizing and Individualizing the Pharmacological Treatment of First-Episode Schizophrenic Patients: Study Protocol for a Multicenter Clinical Trial. Front. Psychiatry.

[10] Penninx B.W.J.H., Lange S.M.M. (2018). Metabolic Syndrome in Psychiatric Patients: Overview, Mechanisms, and Implications. Dialogues Clin. Neurosci..

[11] Werner F.-M., Covenas R. (2017). Long-Term Administration of Antipsychotic Drugs in Schizophrenia and Influence of Substance and Drug Abuse on the Disease Outcome. Curr. Drug Abuse Rev..

[12] Meyer J.M. (2007). Strategies for the Long-Term Treatment of Schizophrenia: Real-World Lessons from the CATIE Trial. J. Clin. Psychiatry.

[13] Ratna V.V.J., Vempadapu M., Kolakota R.K., Mugada V. (2019). Risk of Cardiovascular Disease in Schizophrenia: A Mini Review. Asian J. Res. Pharm. Sci..

[14] Martin-Rodriguez E., Guillen-Grima F., Martí A., Brugos-Larumbe A. (2015). Comorbidity Associated with Obesity in a Large Population: The APNA Study. Obes. Res. Clin. Pract..

[15] Santos Palacios S., Llavero Valero M., Brugos-Larumbe A., Díez J.J., Guillén-Grima F., Galofré J.C. (2018). Prevalence of Thyroid Dysfunction in a Large Southern European Population. Analysis of Modulatory Factors. The APNA Study. Clin. Endocrinol..

[16] Martin-Rodriguez E., Guillen-Grima F., Aubá E., Martí A., Brugos-Larumbe A. (2016). Relationship between Body Mass Index and Depression in Women: A 7-Year Prospective Cohort Study. The APNA Study. Eur. Psychiatry.

[17] Brugos-Larumbe A., Aldaz-Herce P., Guillen-Grima F., Garjón-Parra F.J., Bartolomé-Resano F.J., Arizaleta-Beloqui M.T., Pérez-Ciordia I., Fernández-Navascués A.M., Lerena-Rivas M.J., Berjón-Reyero J. (2018). Assessing Variability in Compliance with Recommendations given by the International Diabetes Federation (IDF) for Patients with Type 2 Diabetes in Primary Care Using Electronic Records. The APNA Study. Prim. Care Diabetes.

[18] Alvarez-Mon M.A., Guillen-Aguinaga S., Pereira-Sanchez V., Onambele L., Al-Rahamneh M.J., Brugos-Larumbe A., Guillen-Grima F., Ortuño F. (2021). Being Born in Winter-Spring and at Around the Time of an Influenza Pandemic Are Risk Factors for the Development of Schizophrenia: The Apna Study in Navarre, Spain. J. Clin. Med..

[19] Guillen-Aguinaga S., Forga L., Brugos-Larumbe A., Guillen-Grima F., Guillen-Aguinaga L., Aguinaga-Ontoso I. (2021). Variability in the Control of Type 2 Diabetes in Primary Care and Its Association with Hospital Admissions for Vascular Events. The APNA Study. J. Clin. Med..

[20] (2012). Real Decreto-Ley 16/2012, de 20 de Abril, de medidas urgentes para garantizar la sostenibilidad del Sistema Nacional de Salud y mejorar la calidad y seguridad de sus prestaciones. Boletín Of. del Estado.

[21] Charlson M.E., Pompei P., Ales K.L., MacKenzie C.R. (1987). A New Method of Classifying Prognostic Comorbidity in Longitudinal Studies: Development and Validation. J. Chronic Dis..

[22] Quan H., Li B., Couris C.M., Fushimi K., Graham P., Hider P., Januel J.-M., Sundararajan V. (2011). Updating and Validating the Charlson Comorbidity Index and Score for Risk Adjustment in Hospital Discharge Abstracts Using Data from 6 Countries. Am. J. Epidemiol..

[23] Haddad P., Brain C., Scott J. (2014). Nonadherence with Antipsychotic Medication in Schizophrenia: Challenges and Management Strategies. Patient Relat. Outcome Meas..

[24] Velligan D.I., Lam Y.-W.F., Glahn D.C., Barrett J.A., Maples N.J., Ereshefsky L., Miller A.L. (2005). Defining and Assessing Adherence to Oral Antipsychotics: A Review of the Literature. Schizophr. Bull..

[25] Lederer D.J., Bell S.C., Branson R.D., Chalmers J.D., Marshall R., Maslove D.M., Ost D.E., Punjabi N.M., Schatz M., Smyth A.R. (2019). Control of Confounding and Reporting of Results in Causal Inference Studies. Guidance for Authors from Editors of Respiratory, Sleep, and Critical Care Journals. Ann. Am. Thorac. Soc..

[26] Hernán M., Robins J. (2020). CausalInference: What If.

[27] Textor J., van der Zander B., Gilthorpe M.S., Liśkiewicz M., Ellison G.T.H. (2017). Robust Causal Inference Using Directed Acyclic Graphs: The R Package ‘Dagitty’. Int. J. Epidemiol..

[28] Dean A.G., Sullivan K.M., Soe M.M. OpenEpi: Open Source Epidemiologic Statistics for Public Health, Versión 3.01. https://www.openepi.com/.

[29] Abidi O., Vercherin P., Massoubre C., Bois C. (2019). Le Risque Cardiovasculaire Global Des Patients Atteints de Schizophrénie Hospitalisés En Psychiatrie Au CHU de Saint-Étienne. Encephale..

[30] Yang X., Chen Y., Wang H., Fu X., Kural K.C., Cao H., Li Y. (2021). Schizophrenia Plays a Negative Role in the Pathological Development of Myocardial Infarction at Multiple Biological Levels. Front. Genet..

[31] Corfdir C., Pignon B., Szöke A., Schürhoff F. (2021). Accelerated Telomere Erosion in Schizophrenia: A Literature Review. Encephale.

[32] Saravane D., Feve B., Frances Y., Corruble E., Lancon C., Chanson P., Maison P., Terra J.-L., Azorin J.-M. (2009). Élaboration de Recommandations Pour Le Suivi Somatique Des Patients Atteints de Pathologie Mentale Sévère. Encephale.

[33] Solmi M., Fiedorowicz J., Poddighe L., Delogu M., Miola A., Høye A., Heiberg I.H., Stubbs B., Smith L., Larsson H. (2021). Disparities in Screening and Treatment of Cardiovascular Diseases in Patients with Mental Disorders Across the World: Systematic Review and Meta-Analysis of 47 Observational Studies. Am. J. Psychiatry.

[34] Kapral M.K., Kurdyak P., Casaubon L.K., Fang J., Porter J., Sheehan K.A. (2021). Stroke Care and Case Fatality in People with and without Schizophrenia: A Retrospective Cohort Study. BMJ Open.

[35] Lemogne C., Blacher J., Airagnes G., Hoertel N., Czernichow S., Danchin N., Meneton P., Limosin F., Fiedorowicz J.G. (2021). Management of Cardiovascular Health in People with Severe Mental Disorders. Curr. Cardiol. Rep..

[36] Lai F.T.T., Guthrie B., Mercer S.W., Smith D.J., Yip B.H.K., Chung G.K.K., Lee K.-P., Chung R.Y., Chau P.Y.K., Wong E.L.Y. (2020). Association between Antipsychotic Use and Acute Ischemic Heart Disease in Women but Not in Men: A Retrospective Cohort Study of over One Million Primary Care Patients. BMC Med..

[37] Chen C.-H., Leu S.-J.J., Hsu C.-P., Pan C.-C., Shyue S.-K., Lee T.-S. (2021). Atypical Antipsychotic Drugs Deregulate the Cholesterol Metabolism of Macrophage-Foam Cells by Activating NOX-ROS-PPARγ-CD36 Signaling Pathway. Metabolism.

[38] Santa Cruz E.C., Zandonadi F.d.S., Fontes W., Sussulini A. (2021). A Pilot Study Indicating the Dysregulation of the Complement and Coagulation Cascades in Treated Schizophrenia and Bipolar Disorder Patients. Biochim. Biophys. Acta. Proteins Proteom..

[39] Saha S., Welham J., Chant D., McGrath J. (2006). Incidence of Schizophrenia Does Not Vary with Economic Status of the Country: Evidence from a Systematic Review. Soc. Psychiatry Psychiatr. Epidemiol..

[40] Hjorth P., Juel A., Hansen M.V., Madsen N.J., Viuff A.G., Munk-Jørgensen P. (2017). Reducing the Risk of Cardiovascular Diseases in Non-Selected Outpatients With Schizophrenia: A 30-Month Program Conducted in a Real-Life Setting. Arch. Psychiatr. Nurs..

[41] Selby P., Vojtila L., Ashfaq I., Dragonetti R., Melamed O.C., Carriere R., LaChance L., Kohut S.A., Hahn M., Mulsant B.H. (2021). Technology-Enabled Collaborative Care for Youth with Early Psychosis: A Protocol for a Feasibility Study to Improve Physical Health Behaviours. Early Interv. Psychiatry.

[42] Fenton A., Sharps P., Kverno K., RachBeisel J., Gorth M. (2021). A 12-Week Evidence-Based Education Project to Reduce Cardiovascular and Metabolic Risk in Adults With Serious Mental Illness in the Integrated Care Setting. J. Am. Psychiatr. Nurses Assoc..

