# Supplementary material for: Schizophrenia and Hospital Admissions for Cardiovascular Events in a Large Population: The APNA Study
Source: J Cardiovasc Dev Dis. 2022 Jan 13;9(1):25. doi: 10.3390/jcdd9010025 (PMC8778060; doi:10.3390/jcdd9010025)
Supplement: Supplementary file 1 [file jcdd-09-00025-s001.zip › Suplementary.pdf]

Table S1. Univariate and multivariate cox regression for hospital admission for cardiovascular events adjusted by Charlson Index.

	Model Adjusted by age and sex		Model Adjusted*	
Variables	HR (95% CI)	p	HR (95% CI)	p
Schizophrenia	1.348 (1.009-1.801)	0.044	1.414 (1.031-1.938)	0.031

HR= Hazard Ratio * Adjusted by Hypertension, Age, Sex, Type 2 Diabetes, Dyslipidemia, CV Antecedents, Smoking, Low Income, Obesity (BMI > 30), and Charlson Index

Table S2. Univariate and multivariate cox regression for hospital admission for cardiovascular events according to adherence to schizophrenia treatment adjusted by Charlson Index

	Model Adjusted by age and sex		Model Adjusted*	
Variables	HR (95% CI)	p	HR (95% CI)	p
Untreated Schizophrenia	1.206 (0.848-1.717)	0.297	2.270 (1.288-4.001)	0.005
Treated Schizophrenia	1,778 (1.071-2.951)	0.026	1.211 (0.829-1.767)	0.322

HR= Hazard Ratio *Adjusted by Hypertension, Age, Ssex, Type 2 Diabetes, Dyslipidemia, CV Antecedents, Smoking, Low Income, Obesity (BMI > 30), and Charlson

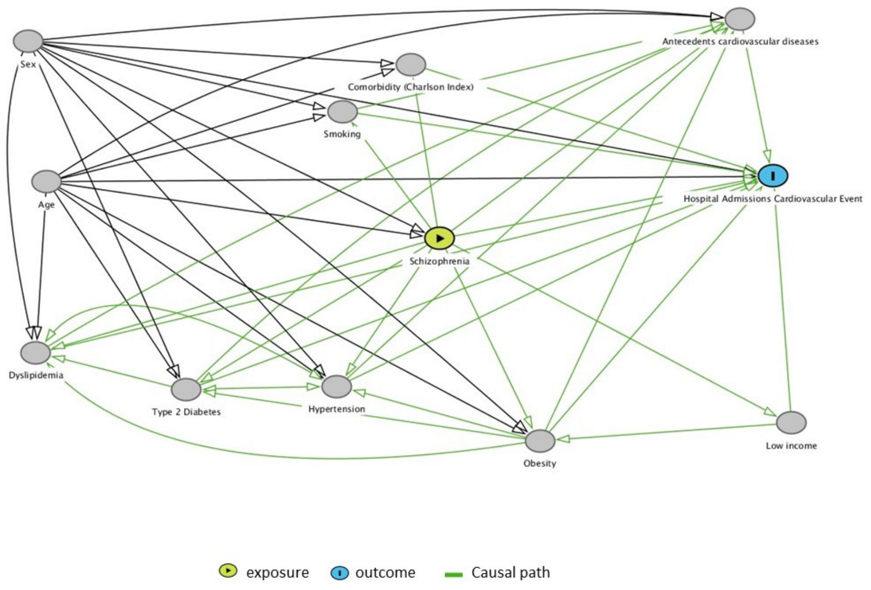

Figure S1. DAG Showing the causal paths from schizophrenia to hospital admission for cardiovascular events
